# Real-time detection of chlorine gas using Ni/Si shell/core nanowires

**DOI:** 10.1186/s11671-015-0729-2

**Published:** 2015-01-28

**Authors:** Dong-Jin Lee, Kwang Heo, Hyungwoo Lee, Joon-Hyung Jin, Hochan Chang, Minjun Park, Han-Bo-Ram Lee, Hyungjun Kim, Byung Yang Lee

**Affiliations:** School of Mechanical Engineering, Korea University, 145 Anam-Ro, Seongbuk-Gu, Seoul 136-713 Korea; Department of Bioengineering, University of California, 261 Donner Lab, Berkeley, CA 94720 USA; Department of Materials Science and Engineering, University of Wisconsin-Madison, 1509 University Avenue, Madison, WI 53706 USA; Department of Materials Science and Engineering, Incheon National University, 119 Academy-ro, Yeonsu-gu, Incheon Korea; School of Electrical and Electronic Engineering, Yonsei University, 50 Yonsei-Ro, Seodaemun-Gu, Seoul Korea

**Keywords:** Nanowires, Self-assembly, Atomic layer deposition, Sensor, Halogen gas

## Abstract

We demonstrate the selective adsorption of Ni/Si shell/core nanowires (Ni-Si NWs) with a Ni outer shell and a Si inner core on molecularly patterned substrates and their application to sensors for the detection of chlorine gas, a toxic halogen gas. The molecularly patterned substrates consisted of polar SiO_2_ regions and nonpolar regions of self-assembled monolayers of octadecyltrichlorosilane (OTS). The NWs showed selective adsorption on the polar SiO_2_ regions, avoiding assembly on the nonpolar OTS regions. Utilizing these assembled Ni-Si NWs, we demonstrate a sensor for the detection of chlorine gas. The utilization of Ni-Si NWs resulted in a much larger sensor response of approximately 23% to 5 ppm of chlorine gas compared to bare Ni NWs, due to the increased surface-to-volume ratio of the Ni-Si shell/core structure. We expect that our sensor will be utilized in the future for the real-time detection of halogen gases including chlorine with high sensitivity and fast response.

**Electronic supplementary material:**

The online version of this article (doi:10.1186/s11671-015-0729-2) contains supplementary material, which is available to authorized users.

## Background

Nanoscale hybrid structures of metals and semiconductors have attracted a lot of attention due to their exotic properties [1,2]. For example, metal-coated semiconducting nanowires (NWs) have demonstrated remarkable optical, mechanical, and electronic properties, enabling their application to areas such as field effect transistors and optical waveguides [3-5]. Biochemical sensors using metal–semiconductor hybrid structures have been also a source of growing interest due to their high sensitivity and integration capability [6-8]. Among metal-based materials, nickel and its oxides have been utilized in the detection of ammonia [9,10], glucose [11], and cigarette smoke [12]. In this paper, we demonstrate the utilization of metal-coated Si NWs with Ni/Si shell/core structures (Ni-Si NWs) as sensor transducers for the detection of chlorine (Cl_2_) gas, which is a toxic halogen gas. It is worth noting that not much study has been performed on Cl_2_ gas sensors using nickel oxide materials except for nickel ferrite structure [13], mainly due to its relatively low sensitivity. In this work, we introduced a NW with a Ni-Si core/shell structure and demonstrated that a nickel oxide-based structure with high surface-to-volume ratio can be used for the room temperature real-time detection of chlorine gas. The sensor transducer was prepared by selective adsorption of Ni-Si NWs on molecularly patterned substrates without any functionalization of the NWs. The molecularly patterned substrates consisted of polar SiO_2_ regions and nonpolar octadecyltrichlorosilane (OTS). The NWs were selectively adsorbed on the polar surface regions, avoiding assembly on the nonpolar OTS regions. To utilize these assembled Ni-Si NWs for practical applications, we demonstrated a sensor for the detection of Cl_2_ gas, which is a toxic material generated during many chemical reactions and manufacturing processes [13-15]. Because it can be dangerous when inhaled, a rapid and reliable detection method of Cl_2_ gas is highly demanded [16]. The utilization of Ni-Si NWs resulted in a much larger sensor response to Cl_2_ gas compared to bare Ni NWs, due to the increased surface-to-volume ratio of the sensor transducer. We expect that our sensor can be used in the future for the real-time detection of halogen gas including chlorine with high sensitivity and fast response.

## Methods

### Preparation of Ni-Si NWs

Single-crystalline Si NWs were first grown via chemical vapor deposition (CVD) process with Au catalysts [17,18]. The surface of CVD-grown Si NWs was usually coated with native oxide, and their surface properties varied depending on the growth condition and degree of surface contamination [18]. The Ni thin film was then deposited by the atomic layer deposition (ALD) process using bis(dimethylamino-2-methyl-2-butoxo)nickel (Ni(dmamb)_2_) as the precursor and NH_3_ gas as the reactant. This resulted in Ni/Si shell/core structures with uniform Ni thickness of 20 nm. The Ni precursor in a steel bubbler kept at 70°C was carried with Ar gas at a rate of 50 sccm into the main chamber. NH_3_ reactant gas was injected to the chamber at a rate of 400 sccm. The substrate was maintained at 300°C. One ALD cycle consisted of 4 s of Ni(dmamb)_2_ precursor exposure, 1 s of Ar purging, 6 s of NH_3_ gas reactant exposure, and 1 s of Ar purging. The saturated growth rate was 0.64 Å/cycle.

### Preparation of Ni NWs

The bare Ni NWs were grown by the electrodeposition method using anodic aluminum oxide (AAO) substrates as templates [19,20]. To explain briefly, a 400-nm silver (Ag) layer was thermally deposited on one side of the AAO filters with a pore size of approximately 80 nm (Synkera Technologies, Longmont, CO, USA). Afterwards, an additional Ag (Techni Silver 1025, Technic. Inc., Anaheim, CA, USA) film was electrochemically deposited at −0.8 V vs Ag/AgCl for 4 C using a potentiostat (Reference 600, Gamry Instruments Inc., Warminster, PA, USA). Ni (Nickel Sulfamate RTU, Technic. Inc.) was then deposited at −0.85 V vs Ag/AgCl for 6 C. This resulted in Ni NWs with an average length of 20 μm. After Ni growth, the Ag was removed with 4:1:1 (*v*/*v*) solution of methanol, hydrogen peroxide, and ammonium hydroxide. The AAO was removed in 3 M sodium hydroxide solution. Finally, the NWs were rinsed repeatedly with deionized water.

### NW self-assembly and device fabrication

A Ni-Si NW solution (approximately 10^7^ ml^−1^) was first prepared by placing the ALD-processed substrate in deionized (DI) water and applying sonication for 2 min. The assembly process of Ni-Si NWs is similar to the previous assembly methods [18]. First, nonpolar and polar regions were created on solid substrates by patterning self-assembled monolayers (SAMs) of OTS on a SiO_2_ substrate. For nonpolar regions, methyl-terminated OTS SAM was patterned via photolithography [18], while leaving some bare SiO_2_ regions. The methyl-terminated OTS SAM worked as a neutral region. The SiO_2_ surface worked as the polar region in DI water due to the hydroxyl groups (−OH) on the SiO_2_ surface. Ni had an isoelectric point pI of 3.5 to 4 in water [21,22], taking a weak negative surface charge in normal DI water (pH 5.5). When the molecularly patterned substrates were exposed to the Ni-Si NW suspension, the NWs were selectively adsorbed onto the polar SiO_2_ regions due to van der Waals force, avoiding the nonpolar regions. The substrate was then rinsed with DI water to remove weakly adhered NWs. For device fabrication, we deposited Ti/Au (10 nm/50 nm) via thermal evaporation followed by a lift-off process to fabricate the metal electrodes.

### Sensing experiment

The sensing experiments were performed using a homemade gas flowing system consisting of a source gas, flow meters, a N_2_ carrier gas, a chamber, and electrical leads. The Ni-Si NW sensor transducer was placed in the closed chamber, and the NW devices were connected to a computer-controlled two-channel sourcemeter (Keithley 2636A, Keithley Instruments Inc., Cleveland, OH, USA) in a two-probe configuration. Then, Cl_2_ gas of known concentrations (5 and 20 ppm) was sequentially injected into the chamber while simultaneously monitoring the current change of the Ni-Si NW and Ni NW devices. The applied voltage bias was 1 V for the Ni-Si NW and 0.01 V for the Ni NW-based sensors.

## Results and discussion

**Figure 1:**
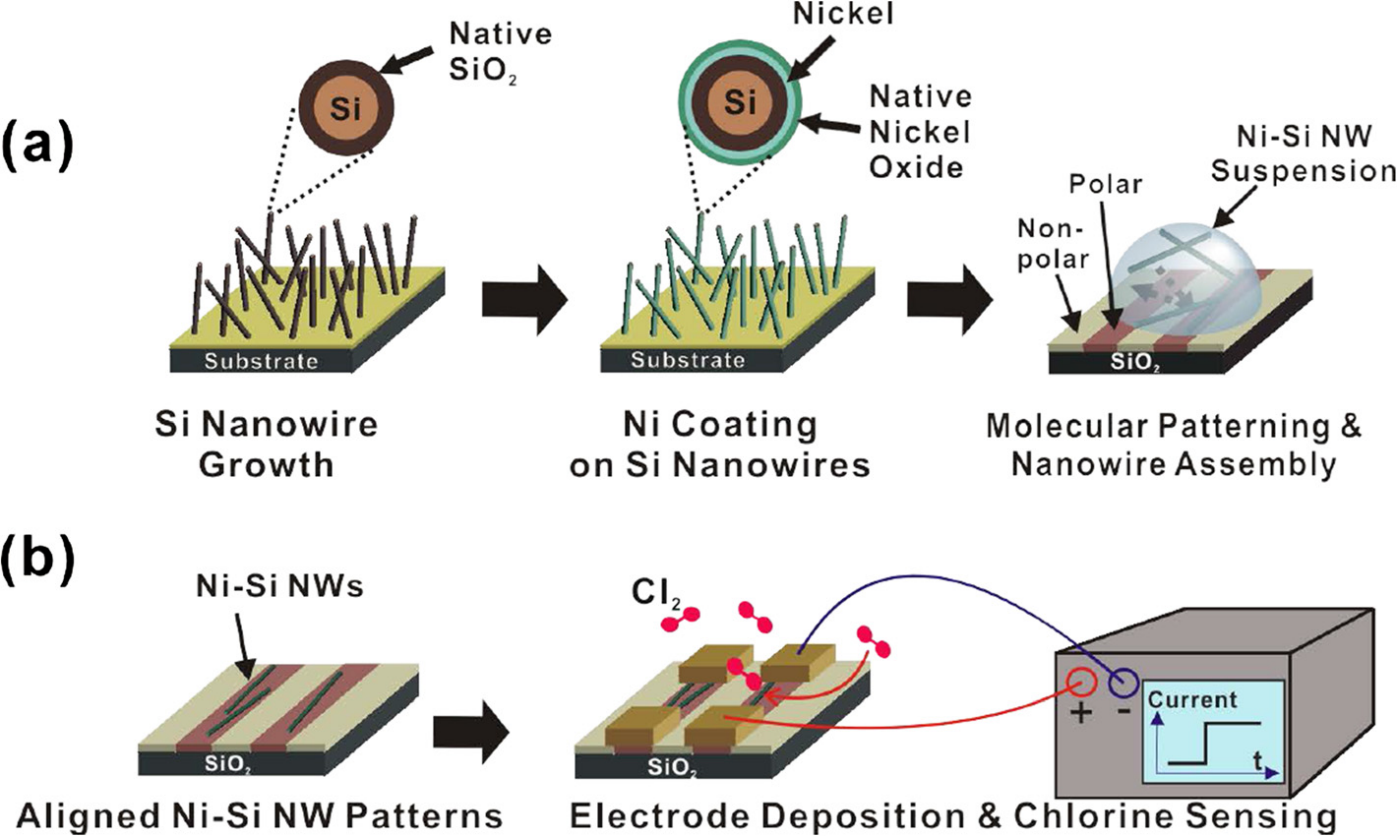
**Schematic diagram describing Ni-Si NW assembly and Cl**_**2**_ **gas detection. (a)** Growth of Ni-Si NWs and their assembly on molecularly patterned substrates. Si NW forest is grown via CVD process (left). Ni coating is performed by ALD process (center). The NWs are sonicated in DI water and drop-cast onto a molecularly patterned substrate (right). The dimensions in the cross-sectional view are not to scale. **(b)** Sensor fabrication and Cl_2_ gas sensing experiments. Electrodes are formed on the assembled Ni-Si NWs and electrical connections are made (left, center). When the sensor is exposed to Cl_2_ gas, conductance change is monitored (right).

Figure 1 shows the schematic description of our sample preparation method and sensing experiments of Cl_2_ gas. Ni-Si NWs were prepared via Si NW CVD growth followed by Ni ALD process [23]. To explain briefly, a Si NW forest was grown using a CVD system [18]. The average length and thickness of the Si NWs after CVD growth were observed as 1 ~ 4 μm and approximately 20 nm, using scanning electron microscopy (SEM; Hitachi S-4300, Hitachi, Tokyo, Japan) [18]. A native oxide layer formed on the Si NW surface under ambient conditions [18]. A 20-nm-thick Ni layer was then coated on the Si NWs by ALD process using Ni(dmamb)_2_ as the precursor and NH_3_ gas as the reactant [23]. This resulted in Ni/Si shell/core structures with an outer diameter of approximately 60 nm. Next, the substrate was dipped into DI water and then mildly sonicated. This process detached the NWs from the substrate, resulting in a Ni-Si NW suspension of concentration approximately 10^7^ ml^−1^ in DI water. The Ni-Si NW suspension was drop-cast on a molecularly patterned SiO_2_ surface consisting of nonpolar OTS regions and polar SiO_2_ regions [18]. When the substrate was placed in a desiccator and left to dry, the solvent evaporated, finally resulting in Ni-Si NW assembled patterns (Figure 1a). For device fabrication, two-probe electrodes (Ti/Au 10/50 nm thick) were formed by photolithography, thermal metal evaporation, and lift-off process. Afterwards, the device was placed in a gas flowing closed chamber and exposed to Cl_2_ gas. The conductance change was monitored using a sourcemeter (Figure 1b).

**Figure 2:**
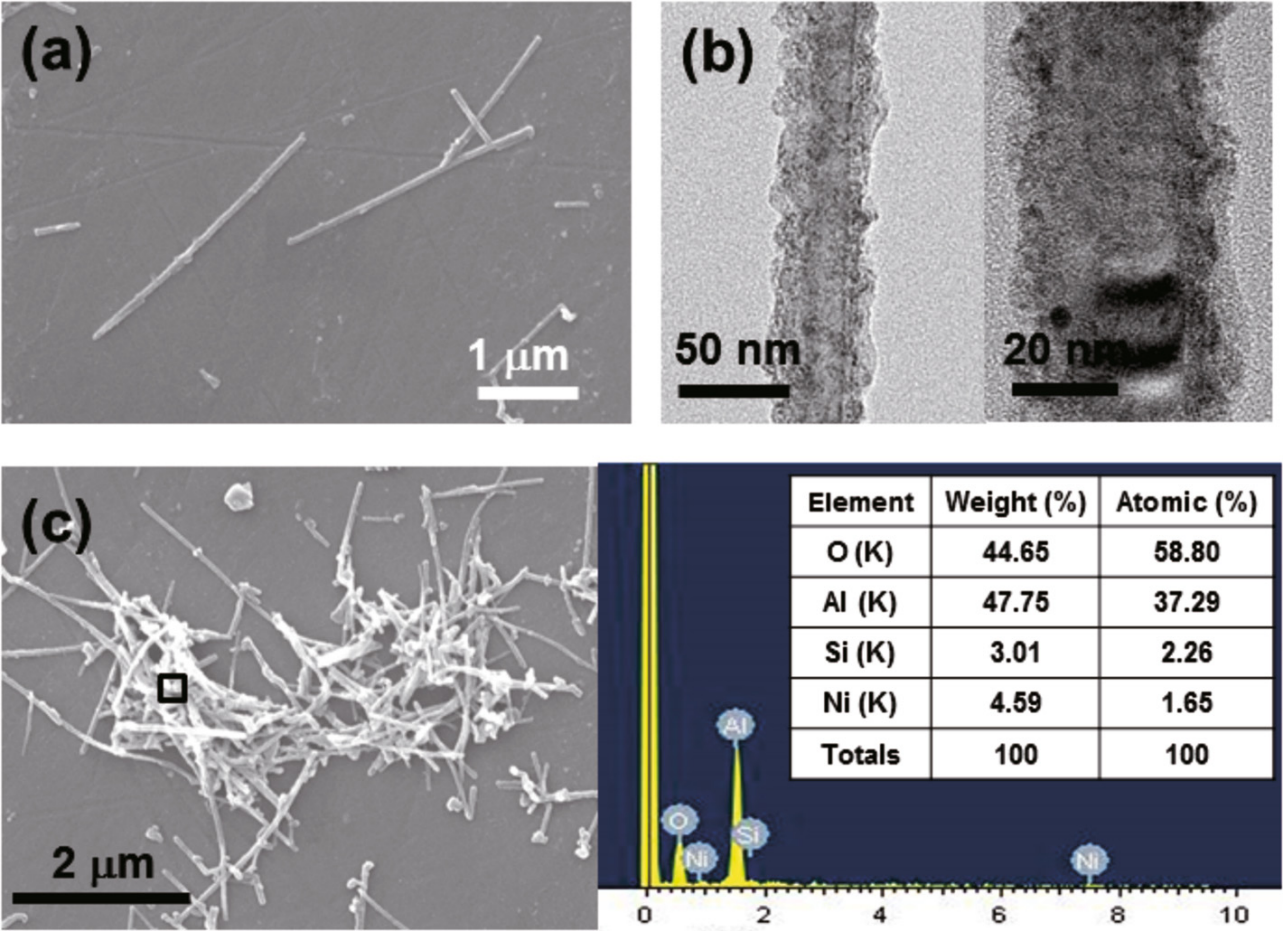
**Electron microscopy images of as-grown Ni-Si NWs. (a)** Scanning electron microscope (SEM) image of individual Ni-Si NWs. **(b)** Transmission electron microscope (TEM) image of a single Ni-Si NW. According to SEM and TEM images, the Ni-Si NWs had a length of 1 ~ 4 μm and a diameter of approximately 60 nm. **(c)** SEM image and EDS data of Ni-Si NWs fixed on a Al_2_O_3_ surface. The rectangle on the SEM image (left) shows the region of EDS analysis. The peaks show the Ni and Si peaks. The Al peak is from the Al_2_O_3_ substrate used for NW fixation.

We characterized the morphology, microstructure, and chemical composition for Ni-Si NWs by SEM, transmission electron microscopy (TEM; JEOL JEM2100F, JEOL, Akishima-shi, Japan), and energy-dispersive spectroscopy (EDS; Hitachi S-4300). The SEM images of the Ni-Si NWs are shown in Figure 2a. The length of the Ni-Si NWs was about 1 ~ 4 μm. The TEM image in Figure 2b shows the shell/core structure of a Ni-Si NW. The Si NW had a diameter of approximately 20 nm, and the average diameter of the Ni-Si NWs was approximately 60 nm after the Ni ALD process. The Ni layer was coated on the Si NW without leaving any vacancy. However, the surface morphology showed a root mean square roughness of 1.95 nm (see Additional file 1: Figure S1). The inner Si core has approximately 1 nm of a native oxide layer [18]. The outer Ni shell of the Ni-Si NWs is also expected to have a few nanometers of native nickel oxide layer [24]. The composition of the Ni-Si NWs was determined from the EDS data as shown in Figure 2c. The NWs were fixed onto a sapphire (Al_2_O_3_) substrate. The EDS data shows an atomic percentage composition of 1.65% for Ni and 2.26% for Si. This is in contrast to bare Ni NWs, which were prepared to compare the sensing properties against Ni-Si NWs (see Additional file 1: Figure S2). The Ni NWs showed no Si peaks, as expected.

**Figure 3:**
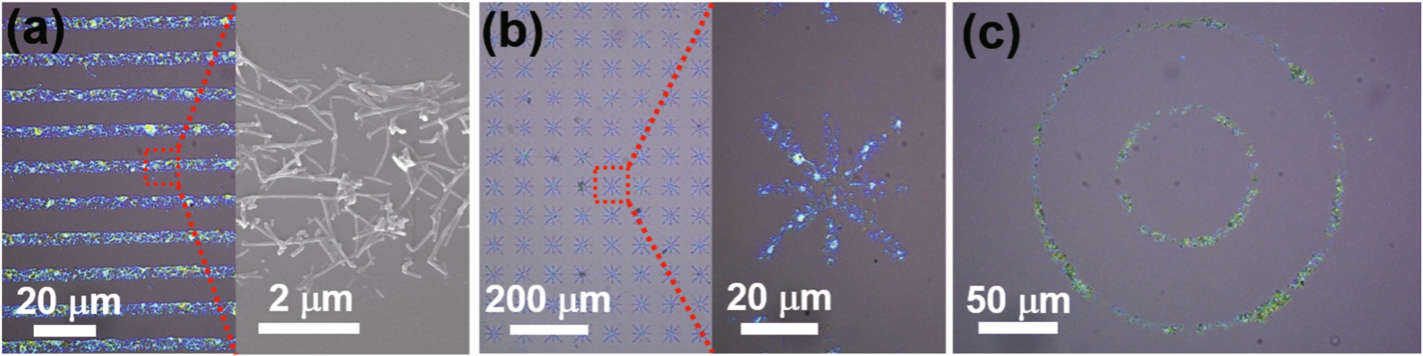
**Selective adsorption of Ni-Si NWs into diverse patterns. (a)** Optical image (left) and magnified SEM image (right) showing the selective adsorption of Ni-Si NWs into line patterns on molecularly patterned SiO_2_ substrates. The NWs were assembled on the polar SiO_2_ regions avoiding the nonpolar OTS regions. **(b)** Optical image (left) and magnified SEM image (right) showing the selective adsorption of Ni-Si NWs into star patterns. **(c)** Optical image showing the selective adsorption of Ni-Si NWs into circle patterns.

After the Ni-Si NW growth, the NWs were dispersed in DI water by mild sonication. Since the adsorbed NWs on solid substrates show no big bundles of NWs as shown in Figure 3a, we can assume that the NWs were almost monodispersed in the solution. The Ni-Si NW solution was drop-cast onto a molecularly patterned SiO_2_ substrate consisting of nonpolar methyl group-terminated OTS and polar hydroxyl group-terminated SiO_2_ regions. The Ni-Si NWs assembled only on the polar SiO_2_ regions, avoiding the nonpolar OTS regions because of the interfacial charge redistribution between the hydroxyl group of the SiO_2_ surface and the Ni NW surface [25,26]. By controlling the width of the OTS pattern, we were able to assemble the NWs to form network structures in diverse patterns (Figure 3). The Ni-Si NWs can be assembled into a line, star, or circle pattern according to the design. This demonstrates the applicability of this molecular force-based assembly method [18] to metal–semiconductor shell/core structures.

**Figure 4:**
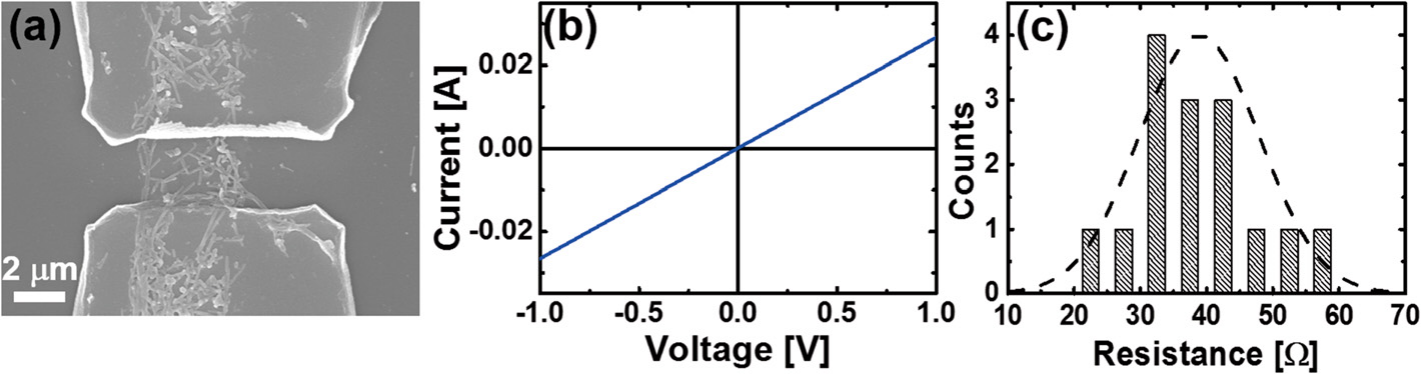
**Electrical properties of networks of Ni-Si NWs. (a)** SEM image of a typical Ni-Si NW network device. **(b)** The IV characteristics of **(a)**. **(c)** The resistance distribution of the Ni-Si NW network devices. The Gaussian fit is indicated in black dashed line.

To characterize the electrical properties of the assembled Ni-Si NWs, we performed additional microfabrication processes to pattern the electrodes (Figure 4). The electrodes were fabricated via photolithography and thermal evaporation of 10-nm/50-nm Ti/Au metal films, followed by a lift-off process. Figure 4a shows the SEM images of a Ni-Si NW network two-probe device. The devices typically showed an ohmic IV curve, as shown in Figure 4b. The resistance distribution of a total number of 15 devices is shown in Figure 4c. The average resistance value was 38.9 Ω, with a standard deviation of 9.1 Ω.

**Figure 5:**
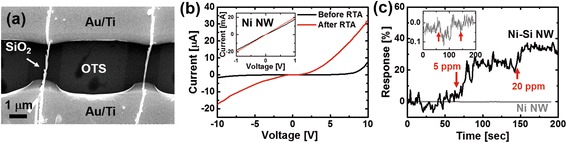
**The sensor response of the Ni-Si NW-based sensor to Cl**_**2**_ **gas. (a)** SEM image of a sensor transducer with two parallel channels of single Ni-Si NWs. **(b)** The IV characteristics of the Ni-Si NWs before and after rapid thermal annealing (RTA) process. For comparison, devices based on bare Ni NWs were also prepared (inset). **(c)** Sensor response of our Ni-Si NW-based device to 5 and 20 ppm of chlorine gas. The Ni-Si NW-based sensor device showed much higher response compared to the bare Ni NW-based device. The apparent noise suppression for the Ni NW device was due to the normalization of sensor response by the initial current value *I*air. The initial current value *I*air for the Ni-Si NW and Ni NW sensor was 0.18 and 152 μA, respectively. The inset shows the expanded view of the sensor response for the Ni NW-based device.

In our Ni-Si NW structure, the Ni shell has increased surface-to-volume ratio compared to bare Ni NWs due to the existence of the Si core. Therefore, we can expect an increased sensor response when Ni-Si NWs are utilized as sensor transducers compared to the bare Ni NWs. Since Ni is known to chemically interact with halogen gas [27,28], we demonstrated the application of our Ni-Si NWs to the detection of Cl_2_ gas, which is a toxic halogen gas [16]. For this purpose, we prepared sensor transducers with single Ni-Si NWs as electronic channels. This was possible by utilizing the same assembly method described above, but by using a ten times diluted solution of NWs with a concentration of approximately 10^6^ ml^−1^. Figure 5a shows two individual single Ni-Si NWs selectively adsorbed on the polar SiO_2_ regions. For comparison, we also prepared devices consisting of single Ni NWs of diameter 80 nm, which were synthesized by electrochemical deposition method (see Methods). Figure 5b shows a typical IV characteristics curve of a Ni-Si NW, and the inset shows that of a Ni NW. The nonohmic behavior in the IV characteristics of a single Ni-Si NW is in contrast to the Ni-Si NW network shown in Figure 3b. This difference in the IV characteristics of these two devices can be explained by the percolation theory, which predicts a threshold areal NW density for the onset of ohmic characteristics from nonohmic behavior for metal/semiconductor shell/core nanostructures [29,30]. The single Ni-Si NW-based device was heat-treated by rapid thermal annealing (RTA) process at 300°C for 20 min to reduce contact resistance. For a Ni-Si NW channel, the resistance was reduced about 250 times after RTA. To perform the Cl_2_ sensing experiment, the Ni-Si NW and the Ni NW sensor transducers were placed together inside a gas flow chamber and then the devices were exposed sequentially to 5 and 20 ppm of Cl_2_ gas. In order to directly compare the performance of the two devices, we prepared sensors with *single* Ni-Si or Ni NW on their channels. The Ni-Si NW and Ni NW devices were applied with 1- and 0.01-V bias voltage, respectively, while monitoring their current change simultaneously. The sensor response here is defined as the ratio of the sensor current change (*I*_gas_ − *I*_air_) after exposure to Cl_2_ gas with respect to its initial value in air (*I*_air_): Response $\left[\%\right]=\frac{I_{\mathrm{gas}}-{I}_{\mathrm{air}}}{I_{\mathrm{air}}}\times 100.$ The sensor response of the Ni-Si NW sensor to Cl_2_ gas of 5 and 20 ppm is shown in Figure 5c. As soon as Cl_2_ gas was injected, the Ni-Si NW sensor showed a response increase within 10 s up to 23% and 34% for 5 and 20 ppm, respectively (Figure 5c, solid black). Compared with other pure metal oxide semiconductor-based sensors, our proposed sensor transducer showed room temperature operation and comparable response time [15,31-33]. Some previous works reported a ppm to sub-ppm detection limit, which is a few orders lower than our work [15,32]. However, we expect that we can improve the detection limit in the future by optimizing several device parameters such as oxide thickness and core/shell dimension. On the other hand, the Ni NW-based sensor transducer showed negligible change in the sensor response (Figure 5c, solid gray). Here, since the conductance of the Ni NWs was generally higher by four orders of magnitude compared to Ni-Si NWs, we applied 0.01 V to the Ni NWs instead of 1 V in order to limit the current through the Ni NWs to comparable levels with Ni-Si NWs. We also tried applying a 0.1-V bias voltage to the Ni NW sensor transducer. However, we observed no change of sensor response (see Additional file 1: Figure S3). This shows that our Ni-Si shell/core structure is more sensitive to external gas stimuli due to an increased surface-to-volume ratio. In terms of sensing mechanism, our Ni-Si NWs showed current increase when exposed to Cl_2_ gas. This current increase is in contrast to bulk Ni film [34], where a current decrease was reported. This difference can be explained by taking into account the role of native nickel oxide (NiO on the Ni shell) [35]. It is known that a native oxide film with thickness of a few nanometers is formed on the Ni surface at room temperature [24]. NiO is a p-type semiconductor with a bandgap energy of 3.6 to 4.0 eV and has been widely utilized as a gas-sensing material with environmental stability [10,36]. Here, we propose that the current increase in our sensors is due to the depletion of electrons caused by the replacement of mobile surface oxygen in the native NiO region with the adsorbed Cl_2_, resulting in the increase of hole concentration [13,35,37]. A further study of the effect of intentional oxidation on the Ni-Si NWs to increase the sensitivity to Cl_2_ gas will be performed in the future.

## Conclusions

We have demonstrated the selective adsorption of Ni-Si NWs on molecularly patterned substrates and their application to sensors for the detection of Cl_2_ gas. The Ni-Si NWs have a larger surface-to-volume ratio compared to that of Ni NWs, which makes them more advantageous in detecting Cl_2_ gas. The Ni-Si NW sensor showed the real-time detection to Cl_2_ gas with high sensitivity and fast response time. We expect that our Ni-Si NWs can be utilized in the future as an integrated platform for sensor applications.

## Additional file

Additional file 1: **Supplementary material.**
