# Supplementary material for: Real-time detection of chlorine gas using Ni/Si shell/core nanowires
Source: Nanoscale Res Lett. 2015 Jan 28;10:18. doi: 10.1186/s11671-015-0729-2 (PMC4314467; doi:10.1186/s11671-015-0729-2)
Supplement: Supplementary file 1 — Supplementary material. [file 11671_2015_729_MOESM1_ESM.pdf]

[Supplementary Material]

Real-time Detection of Chlorine Gas using Ni/Si Shell/Core Nanowires

Dong-Jin Lee[1‡], Kwang Heo[2‡], Hyungwoo Lee[3], Joon-Hyung Jin[1], Hochan Chang[1], Minjun Park[1], Han-Bo-Ram Lee[4], Hyungjun Kim[5], Byung Yang Lee[1]*

[1] *School of Mechanical Engineering, Korea University, Seoul 136-713, Korea*

[2] *Department of Bioengineering, University of California, Berkeley, CA 94720, USA*

[3] *Department of Materials Science and Engineering, University of Wisconsin-Madison, Madison, WI 53706, USA*

[4] *Department of Materials Science and Engineering, Incheon National University, Incheon, Korea*

[5] *School of Electrical and Electronic Engineering, Yonsei University, Seoul, Korea*

KEYWORDS: Nanowires, Self-Assembly, Atomic Layer Deposition, Sensor, Halogen Gas.

‡These authors contributed equally.

*Corresponding Author: blee@korea.ac.kr

Surface Roughness of the Ni-Si NWs

To obtain the line roughness of the Ni-Si NW, the surface topography was measured by atomic force microscopy (AFM, NTEGRA Prima, NT-MDT) as shown in Fig. S1. From height profile in Fig. S1b, we calculated root-mean-square roughness as follows: $R_q = \sqrt{\frac{1}{N}\sum_{j=1}^{N} h_j^2}$, where N is the total number of line points, and h_j is the representative height of each point.

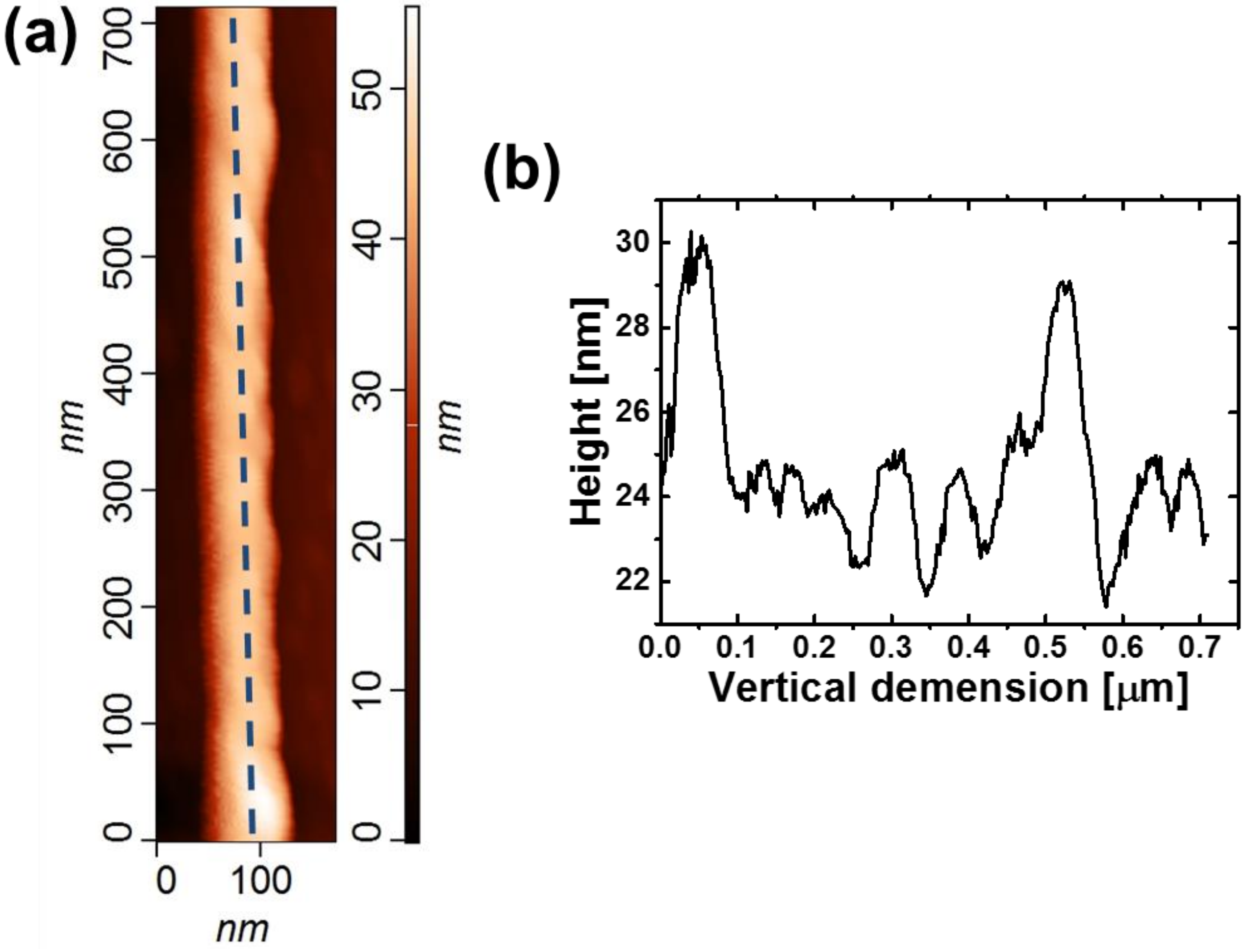

Figure S1. AFM image (a) and height profile (b) of a Ni-Si NWs.

Characterization of the morphology, microstructure, and chemical composition for Ni-Si NWs

For comparison with the Ni-Si NWs, we prepared bare Ni NWs using electrodeposition method in AAO templates. Afterwards, we characterized the morphology, microstructure, and chemical composition of the Ni NWs by SEM, TEM, and EDS. Figure S2a,b show the SEM and TEM image of the Ni NWs, respectively. The length and diameter of the NW was about 20 μm and 80 nm, respectively. From EDS data shown in Fig. S2c, we observed no Si peaks form the Ni NW, as expected.

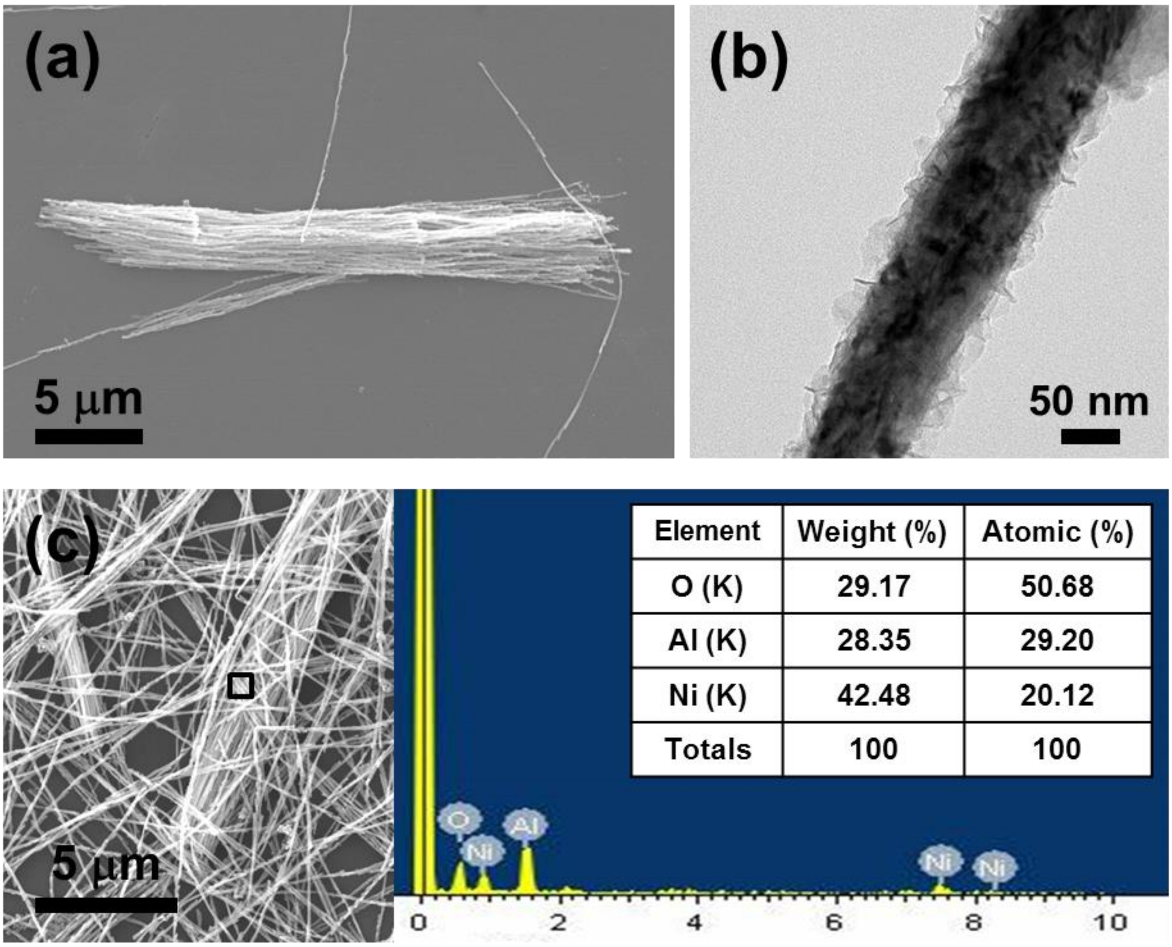

Figure S2. The electron microscopy images of as-grown Ni NWs. (a) The SEM image of a bundle of Ni NWs. (b) TEM image of a single Ni NW. According to SEM and TEM images, the Ni NWs had ~20 um length and ~80 nm diameter. (c) SEM image and EDS data of Ni NWs fixed on Al_2O_3 surface. The Ni NWs showed no Si peaks, as expected. The Al peak is due to Al_2O_3 substrate used for NW fixation.

Sensor response of a Ni NW-based sensor at 0.1V bias voltage

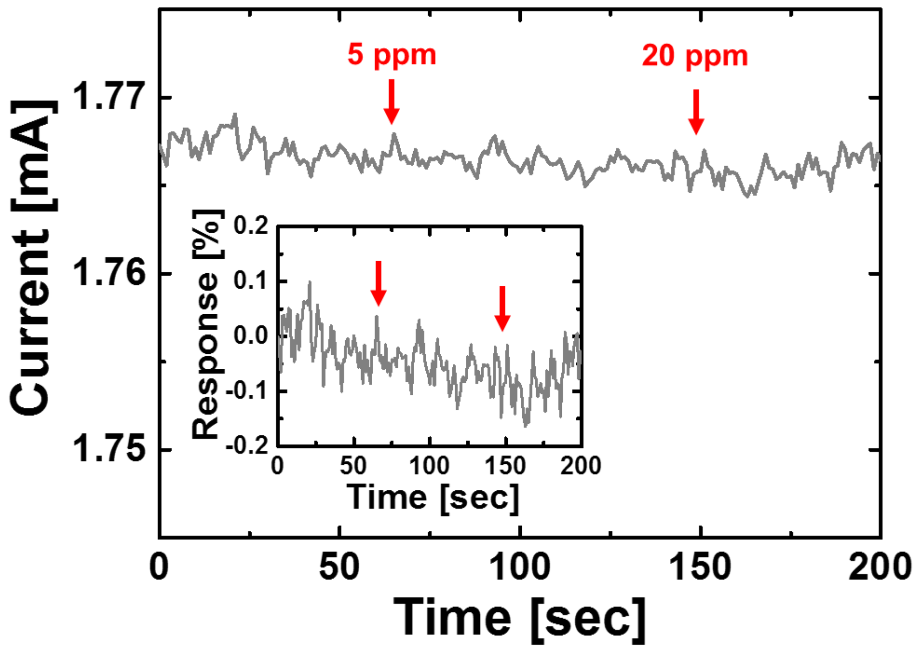

Figure S3. Current variation and sensor response (inset) of our Ni NW-based device to 5 ppm and 20 ppm of chlorine gas at 0.1V bias voltage. The red arrows represent the exposure moment to different concentrations of Cl_2 gas.
